# Comprehensive breast MRI: an update

**DOI:** 10.1186/1470-7330-14-S1-O39

**Published:** 2014-10-09

**Authors:** Sarah J Vinnicombe, Guiseppe Petralia

**Affiliations:** 1 Division of Imaging and Technology, Medical Research Institute, Ninewells Hospital Medical School, University of Dundee, Dundee, DD1 9SY, UK; 2 Department of Radiology, European Institute of Oncology, Via Ripamonti 435, 20141 Milan, Italy

## 

With increasing use of clinical breast MRI and a plethora of novel techniques and sequences for lesion characterisation, a standardised approach to lesion description and reporting is increasingly important. This facilitates meaningful research and audit, particularly in the context of multicentre studies.

To this end the American College of Radiology has recently published the 5^th^ edition of the BI-RADS lexicon, with revision of the MRI section including addition of new descriptors, modification of others and deletion of some that were rarely helpful in practice, such as ‘stippled’ enhancement [1]. Categories for fibroglandular volume are now a to d, in line with the descriptors for mammography, thus avoiding confusion with assessment category. The degree of background parenchymal enhancement is now purely descriptive, from none/minimal to marked. A new descriptor is clustered rim enhancement (usually indicating DCIS). Non mass-like enhancement becomes non mass enhancement, whereas ‘ductal’ enhancement becomes linear. Overall the effect is of simplification and consistency with the mammography and ultrasound lexicons.

The lexicon highlights the importance of a structured report, including the indication, scan technique, salient findings and critically, an overall assessment with a clear recommendation for further management. This approach has been validated in a number of studies correlating the assessment category with histopathological findings and/or long-term follow-up [2,3].

Use of a standardised lexicon, modified in the light of available evidence, has obviated some of the interpretative challenges of breast MRI. Use of supplemental diffusion weighted imaging may also be helpful [4]. Nonetheless, technical and clinical challenges remain. Artefacts are problematic, especially at 3T [5], and medical physics support is essential for good quality diagnostic scans, especially at higher field strength.

Most importantly, it should be remembered that to date, despite the recognised superiority of breast MRI over any other modality for breast cancer detection and local staging, there is no hard evidence for improved patient outcomes. Two randomised controlled trials and seven comparative cohort studies have not shown any benefit for preoperative MRI in terms of re-excision rates; rather, there is a trend to higher mastectomy rates [6]. No convincing beneficial effects have been demonstrated in terms of in-breast tumour recurrence rates, disease-free survival or overall survival. Regarding assessment of response to neoadjuvant chemotherapy, MRI correlates better than mammography or ultrasound with final pathology, but false positive and negative studies are frequent, with underestimation of the amount of residual disease in up to 30% and overestimation in around 20% of cases [7,8]. Though evidence is accruing that MRI may be helpful in early response assessment, this is by no means standardised and is heavily dependent on many factors, not least tumour immunophenotype. Finally, though screening breast MRI for women at high risk has approximately double the sensitivity of mammography with encouraging stage shift and high rates of node negativity, there is as yet no evidence of a reduction in breast cancer mortality [9,10].

This workshop will provide an update on the BI-RADS lexicon and indications for breast MRI, with expert tuition in hands-on analysis of case studies.
